# DFT Study of the Stability and Electronic Properties of Ni-Doped Defected (6,0) and (8,0) Single-Walled Carbon Nanotubes

**DOI:** 10.3390/ma17246236

**Published:** 2024-12-20

**Authors:** Valeria Orazi, Rubén Eduardo Ambrusi, Alejandro Morelli, Alfredo Juan, Jorge Mario Marchetti

**Affiliations:** 1 Instituto de Física del Sur (UNS-CONICET), Av. L. N. Alem 1253, Bahía Blanca B8000CPB, Argentina; valeria.orazi@uns.edu.ar; 2 Departamento de Ingeniería Eléctrica y Computadoras, Universidad Nacional del Sur (UNS), Av. L. N. Alem 1253, Bahía Blanca B8000CPB, Argentina; 3 Departamento de Física, Universidad Nacional del Sur (UNS), Av. L. N. Alem 1253, Bahía Blanca B8000CPB, Argentina; ale.g.morelli@gmail.com; 4 Faculty of Science and Technology, Norwegian University of Life Sciences, Drøbakveien 31, 1430 Ås, Norway; jorge.mario.marchetti@nmbu.no

**Keywords:** SWCNT, vacancy, Ni, adsorption, DFT

## Abstract

The interaction of Ni with (6,0) and (8,0) zigzag carbon nanotube exterior surfaces containing two vacancies was studied using density functional theory (DFT). A two-vacancy defect was analysed in order to anchor Ni, and the pristine nanotube was also considered as a reference for each chirality. The adsorbed Ni stability and the nanotube’s geometry and electronic structure were analysed before and after the adsorption. We compared calculations performed using a general gradient functional with those conducted using two semi-classical dispersion methods to assess the van der Waals forces (PBE-D2 and PBE-D3). In addition, the inclusion of the Hubbard parameter for the correction of Ni d electron self-interaction energy was included, and we evaluated energy and electronic structure changes through atomic-level calculations. Adsorption energy, the density of states, and the charge distribution were obtained to establish the Ni binding on the defective nanotube’s dominating mechanisms. The effect of curvature and applied functional influence was also considered. Furthermore, a bonding analysis was performed to complement our comprehension of the interaction between Ni and the nanotube surfaces. The electronic results show that Ni-doped two-vacancy (6,0) and (8,0) carbon nanotubes can be applied for the development of low-resistance contact materials and spintronic devices, respectively.

## 1. Introduction

Carbon nanotubes (CNTs) are one-dimensional carbon structures with sp^2^ hybridisation in their carbon bonds resulting from the rolling up of graphene planes, an attribute which confers on them extraordinary mechanical properties [1]. Also, they possess a great variety of properties like high thermal conductivity, good electrical conductivity, a high melting point, low density (1.2–2.6 g/cc), and a large surface area (∼1000 m^2^/g) [2]. These physical chemical properties make this kind of carbon material useful for many applications. For instance, it is an ideal additive for use in the fabrication of anode and cathode electrodes for Li-ion batteries with improved electrochemical characteristics, such as energy conversion and storage capacities [3]. Another promising use of CNTs is the sensing of gases produced by the burning of fossil fuels, including NO, NO_2_, CO, CO_2_, and SO_2_. These gases pollute the environment and cause harmful effects on human health. Additionally, in the field of sensors, Yusfi et al. used DFT [4] to study the adsorption of C_2_H_2_ and C_2_H_4_ gas molecules on Nidoped CNTs. Regarding fuel cells, transition metal (TM) and nitrogen co-doped carbide-derived carbon/carbon nanotube composites have been prepared for use as cathodes in anion-exchange membrane fuel cells [5]. CNTs doped with transition metals (TMs) show good properties for the capture of gases like CO, CO_2_, N_2_O, and CH_4_ and are used to reduce pollution [6]. Moreover, TM nanometre clusters encapsulated in nitrogen-doped CNTs were used for efficient CO_2_ capture. In the electronic field, the exploration of the properties of TMs in CNTs resulted in the development of promising materials for spintronic devices [7] and contact with low and high levels of resistance [8]. The study of the adsorption of atoms on nanotube surfaces is essential to achieve low-resistance ohmic contact with nanotubes, to produce nanowires with controllable sizes, and to fabricate functional nanodevices.

Doping constitutes one of the most used techniques to modify electronic structures and, as a result, the properties of carbon materials. Some examples and other applications have already been produced and are available in the literature. For instance, in the case of CNTs, Abbasi et al. [9] study the appropriate combination of N and B dopants for the detection and removal of CO gas. Other works explore different possibilities, such as the use of N-doped single-walled carbon nanotubes (SWCNTs) for heat transfer applications and B, N, or P doping for quantum capacitance, with significant importance for the development of supercapacitors in commercial use [10,11]. Additionally, electrical and mechanical properties are commonly modified through the use of dopants such as iodine [12]. An interesting route to achieve the desired physical–chemical characteristics, such as electrical conductivity, mechanical properties, catalyst performance, and sorption ability, is the use of TMs as dopants [13,14,15,16,17]. For instance, the single-walled carbon nanotube doped with Pt was effective in the adsorption of molecules such as glucose and carbonyl sulphide (COS) [18,19].

Regarding TM atoms, Ni doping on CNTs was widely studied from an experimental and theoretical point of view because of its good performance in many processes, such as in the sensing of SF_6_, CO, and NO; the development of low-weight hydrocarbons; hydrogen adsorption and storage; oxygen adsorption and dissociation; CO_2_ adsorption and activation; and the production of single-atom catalysts (SACs) [20,21,22,23,24,25,26]. All these outstanding properties could have a potential impact in many real-world applications, such as the development of new materials for hydrogen storage that can be used as fuels in cars; the detection of substances like SF_6_, which are important as electric insulators for high-power tension transformers; and the activation of CO_2_, which is fundamental in the catalysis field and participates in the process of many important reactions, such as the catalytic hydrogenation of CO_2_ for methane production. However, the models used for the evaluation of the Ni and SWCNT interaction usually carry out doping with Ni through substitutions or adsorption on pristine or doped single-walled carbon nanotubes (SWCNTs) [20,21,22,23,24,25,27] and are focused on the evaluation of the interaction between a molecule of interest, such as SO_2_, NO, CO_2_, or H_2_, and decomposition products of insulated SF_6_ during discharge [24,27,28,29,30,31]. Another example of this kind of study is the mentioned DFT evaluation of Ni-doped (10,0) SWCNTs to sense C_2_H_2_ and C_2_H_4_ gases [4]. 

Theoretical works that mainly analyse the interaction between Ni and SWCNT were performed, also considering many other TMs, but for (5,5) chirality and with only a single vacancy and without defects [7,32,33]. Additionally, the inclusion of 3D TMs like Co, Fe, and Ni in SWCNTs was studied via a first-principles approach on (4,4) and (8,8) chiralities. We examined the effect of curvature on the interaction but without the inclusion of vacancy defects [34]. Regarding SWCNT (n,0) zigzag nanostructures, the Ni interaction with the surface was studied using pristine (10,0) SWCNTs [35]. For the (6,0) SWCNT chirality, we analysed the interactions with TMs, but only for Cu, Ag, and Au [36]. To the best of our knowledge, the analysis of the Ni stability and its binding properties on SWCNT with two vacancies was not performed using a systematic approach.

The (8,0) SWCNT is a semiconductor SWCNT with high curvature. This fact means that the (8,0) SWCNT can be applied in several areas, such as electronic devices, hydrogen storage, the production of cathodes for lithium-ion batteries, and gas sensing. It has been demonstrated that the conductivity of semiconducting SWCNTs undergoes a rapid change in response to the adsorption of the gas. Furthermore, the adsorption of gases is enhanced when the SWCNTs are decorated with impurities (e.g., transition or alkali metals) or exhibit structural defects. The (8,0) SWCNT has been the subject of extensive study and presents a number of interesting physical and chemical properties, due to its status as one of the smallest-diameter semiconductor carbon nanotubes [37,38]. These studies demonstrate a substantial alteration in the electronic configuration of the CNT after the adsorption of CO, O_2_, and H_2_ molecules. For these reasons, we selected (8,0) SWCNT as our support, and because its active electronic properties could prevent changes caused by interaction with the environment [39]. Given that the (8,0) CNT has a diameter of 6.37 Å, we selected the (6,0) CNT for comparison, given its smaller diameter of 4.76 Å [40]. Additionally, the isolated (6,0) SWCNT is a metallic material, while the isolated (8,0) SWCNT is a semiconductor.

A recent publication addresses the study of multi-vacancy formation, the most stable configuration and chirality effect of bare SWCNTs [41], which was considered an excellent starting point for further studies of this type of structure with Ni. The present work addresses the lack of a systematic study that evaluates the mechanisms and interactions participating in the adsorption of Ni over the exterior surface of SWCNT in depth. To this end, we performed a DFT investigation of the interaction of a Ni atom with two vacancies on (6,0) and (8,0) SWCNTs. Furthermore, we include the adsorption of Ni on pristine (6,0) and (8,0) SWCNTs in order to achieve benchmark results. Our aim is to elucidate the stability, adsorption geometry, curvature effects, and electronic and magnetic properties of these systems. Also, a detailed examination of the model’s sensitivity to semi-classical dispersion forces and the impact on the adsorption of localised Ni atomic orbitals, treated with and without the Hubbard parameter scheme, is conducted.

## 2. Computational Method

The calculations were performed using the DFT method, implemented in the Vienna Ab Initio Simulation Package (VASP, vs. 6.4.2) [42,43]. To solve the Kohn–Sham equations, a variational approach was employed utilising a periodic supercell method, whereby the wave functions were expanded in a plane–wave basis set. In order to evaluate the quantum effects and the pseudopotential that could be used to replace the core states density, a Perdew, Burke, and Ernzerhof (PBE)-gradient-corrected approximation functional [44] and a projector-augmented wave (PAW) method [45,46] were used. Also, to incorporate solutions with reasonable kinetic energy, a cutoff energy of 500 eV was established to restrict the plane–wave basis set expansion, utilising a gamma-centred Monkhorst–Pack scheme [47]. This value is 20% and 46% larger than the maximum cutoff energy corresponding to the pseudopotential employed for Ni and C atoms, respectively. In all the calculations, spin polarisation was accounted for, and a Gaussian smearing of 0.05 eV was employed.

The geometry optimisations were conducted using a 1 × 1 × 1 and 1 × 1 × 3 k-point grids for the integration over the Brillouin zone, with the objective of minimising the total energy of the supercell. This was achieved through the application of a conjugated gradient algorithm to facilitate the relaxation of ions [48]. The selection of the geometry optimisations is based on the length of all the lattice vectors of the supercell, which will be described in this Section. A tolerance criterion of 10^−4^ eV was applied for the electronic minimisation convergence, and a magnitude of 0.01 eV/Å was used for the forces on each ion in the structure relaxation. The k-point grids used were sufficient to obtain well-converged calculations with variations on the adsorption energies of the order of 10^−3^ eV. Grimme’s DFT D2 and D3 with zero damping methods were employed to account for the PBE functional the van der Waal (vdW) dispersion interactions, which were optimised for several DFT functionals [49,50]. To perform the relaxation calculations of the structure and obtain the equilibrium positions of the atoms, the PBE-D2 and PBE-D3 functionals were employed. Nevertheless, only minor alterations were observed in the equilibrium atoms positions of the atoms, with bond distances exhibiting minimal variation in the first or second decimal place. 

**Figure 1:**
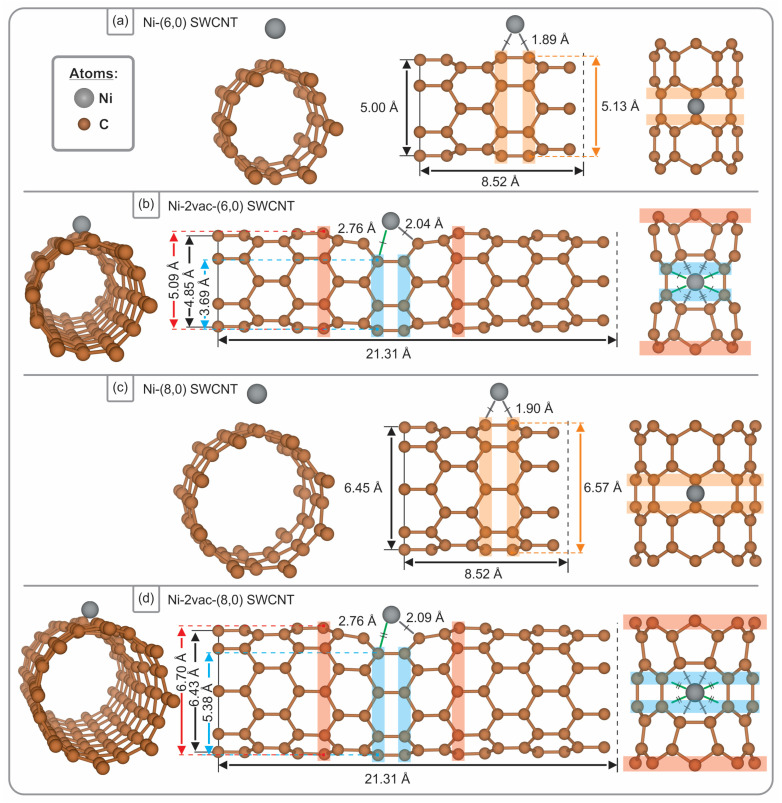
This Figure presents front, lateral, and top views of the optimised structures for a Ni atom adsorbed on (**a**) pristine (6,0) SWCNT, (**b**) di-vacancy (6,0) SWCNT, (**c**) pristine (8,0) SWCNT, and (**d**) di-vacancy (8,0) SWCNT. The optimisation was performed with PBE-D2 functional.

This study is primarily based on two models representing the surface of a SWCNT. A straightforward model was devised to facilitate a comparative analysis of the adsorption energies, electronic structure, bonding calculations, and effective potentials associated with Ni adatom adsorption on pristine (6,0) and (8,0) SWCNTs. In order to model the pristine SWCNT, a periodic supercell of 8.52 Å along the z-axis was constructed, comprising 48 and 64 atoms for the (6,0) and (8,0) types, respectively (see Figure 1a,c). A 20 Å vacuum distance was maintained perpendicular to the nanotube axis. Additionally, a defected SWCNT supercell was constructed based on the methodology proposed by Jia et al. [41], comprising 120 and 160 carbon atoms for the (6,0) and (8,0) type zigzag tubes, respectively. In this second model, a length of 21.3 Å along the SWCNT axis was set, represented by the z-axis and considered periodic (see Figure 1b,d). To avoid interactions between defect images along the perpendicular directions (x and y axes) of the supercell, a vacuum extension of 20 Å was introduced. Furthermore, two atoms were removed along the z-axis direction in the SWCNTs, which has been identified as the most stable di-vacancy configuration in previous research [41]. The optimised defected SWCNTs served as the basis for evaluating the interactions between Ni and SWCNTs. The vacancy formation energy ($E_{formation}$) was calculated using the following Equation:

(1) $$E_{formation}=(E_{vac}+2\mu_{c}-E_{pristine})/2$$

where $E_{vac}$ and $E_{pristine}$ are the total energy of a SWCNT with two vacancies and without defects, and $\mu_{c}$ is the chemical potential of a single C atom, which was calculated as the magnitude of $E_{pristine}$ divided by the number of atoms of the entire pristine SWCNT system.

The vacancy formation energy values of 1.35 eV, 1.74 eV (DFT-D2) and 1.39 eV, 1.76 eV (DFT-D3) obtained for (6,0) and (8,0), respectively, are in good agreement with the theoretical results, with differences in the order of 0.3 and 0.1 eV [41]. Furthermore, the impact of the Hubbard parameter on the energy and electronic states of the localised Ni d electrons was evaluated through a DFT + U approach, in accordance with methodology proposed by Dudarev et al. [51]. This is based on the Hubbard model, which describes the two-electrons potential term on the assumption that the Coulomb interaction is significant between two electrons occupying the same site (orbital). Consequently, these electrons interact through a constant potential energy, U. In this approach, only the difference U_eff_ = U − J is taken into account, where U and J parameters express the influence of the effective on-site Coulomb interactions and on-site exchange interactions, respectively. In the present work, a value of U_eff_ = 2 eV is employed, which, as evidenced in previous studies [52,53], effectively represents the electronic behaviour due to the localisation effect of d orbitals of TMs. The results obtained with the two dispersion methods and without U_eff_ parameter were compared to establish the sensitivity of the system to these effects.

Following the optimisation process, the adsorption energy of Ni atom on SWCNT ($E_{ads}$) was calculated using the following Equation:

(2) $$E_{ads}=E_{Ni-SWCNT}-E_{SWCNT}-E_{Ni}$$

In this Equation, $E_{Ni-SWCNT}$ and $E_{SWCNT}$ represent the total energy of the SWCNT with and without adsorbed Ni, respectively. Additionally, $E_{SWCNT}$ represents the energy of the clean SWCNT, including or excluding any defects. $E_{Ni}$ represents the energy of an isolated Ni atom, for the most stable triplet ground state. A negative value of this Equation indicates a stable configuration.

The electronic structure was analysed by performing a number of calculations, including the calculation of the density of states (DOS), partial density of states (PDOS), Bader charges [54], overlap population (OP), and bond order (BO) values, in order to gain insight into the bonding. OP and BO calculations were performed using the Chargemol code [55,56,57].

The charge density difference was computed using the following Equation:

(3) ∆ρ=ρ[Ni−(n,0) SWCNT]−ρ[(n,0) SWCNT]−ρ[Ni]

where the first two terms, ρ[Ni−(n,0) SWCNT] and ρ[(n,0) SWCNT], represent the electron densities of the systems (*n*,0) SWCNT with or without defects after and before the Ni adsorption, respectively. These terms employ the atom positions for the relaxed Ni-(*n*,0) SWCNT structures. The term ρ[Ni] represents the density of Ni at the adsorption position, without the (*n*,0) SWCNT. The calculations were performed for *n* equal to 6 and 8.

## 3. Results and Discussion

### 3.1. Geometry Structure, Adsorption Energy, and Magnetization

Figure 1 illustrates the optimised structures after the interaction with a Ni atom. Despite the Ni-C bond distance being lower for the adsorbed Ni atom on the pristine SWCNT, at 1.89 Å and 1.90 Å ((6,0) and (8,0), respectively) compared to 2.04 Å and 2.09 Å (for the nearest C atoms) on SWCNTs with di-vacancies ((6,0) and (8,0), respectively), there is a larger coordination when the vacancy defect is present. The values obtained for the pristine SWCNT are in good agreement with those reported in the literature for chiralities (5,0), (5,5), and (8,0) with PBE and also with local density approximation functionals [25,27,58], falling within the range of 1.91–1.98 Å. To the best of our knowledge, there are no reported data concerning Ni-C bond distances for Ni interaction with two vacancies. Xiao et al. employed DFT calculations to demonstrate that functionalised carbon nanotubes containing Ni, Rh, or Pd exhibit enhanced hydrogen adsorption properties [27]. Chen et al. investigated the adsorption of several TMs adsorbed on CNTs [33]. The length of the TM–carbon bond decreases from Sc to Fe and then increases from Co to the last element in this row, Zn. The Cu- and Zn-doped SWCNTs exhibit exceptionally long TM–carbon bonds. In the case of Ni-, Pd-, and Pt-doped SWCNTs, the Pd-C bond is observed to be longer than the Pt-C bond. Furthermore, both of these bonds are still longer than the Ni-C bond, which can be attributed to the phenomenon of lanthanide contraction. The V-, Cr-, and Mn-doped SWCNTs exhibit comparable TM–carbon bond lengths, due to the formation of chemical bonds [33]. Our Ni-C distance is consistent with the findings of a previous systematic study of the adsorption of single atoms on the (8,0) and (6,6) SWCNTs [58].

In the case of Ni adsorbed at the vacancy site (see Figure 1b,d), it can be observed that the preferred position is at the centre. However, based on the observed structure, the bond between Ni and the carbon atoms in the pentagons is more favourable than that with those in the hexagons, resulting in Ni remaining in closer proximity to the pentagons.

In the case of the pristine (6,0) SWCNT (Figure 1a), the adsorption of a nickel (Ni) atom at the bridge site results in an increase in the diameter of the SWCNT. The average diameter increases from 4.86 Å to 5.00 Å (+2.88%), and the diameter of the rings in closest proximity to the Ni atom, highlighted in orange in the figure, rises from 4.86 Å to 5.13 Å (+5.56%). A comparable pattern is evident when Ni adsorbs onto the pristine (8,0) SWCNT, wherein the bridge site is once again the most favourable (Figure 1c). This results in an increase in the average diameter from 6.35 Å to 6.45 Å (+1.57%) and in the diameter of the rings closest to the Ni atom, highlighted in orange, from 6.35 Å to 6.57 Å (+3.46%). The bridge site has been identified as the preferred site for pristine SWCNTs in previous studies [25,58]. Additionally, previous studies [59,60] have reported a diameter of 6.37–6.38 Å for the pristine (8,0) SWCNT, which aligns closely with our findings.

With regard to the (8,0) SWCNT with a di-vacancy, the largest diameter is observed in the carbon rings highlighted in red in Figure 1d, which increases from 6.61 Å before Ni adsorption to 6.70 Å subsequently (+1.36%). Furthermore, the average diameter of this SWCNT also exhibits an increase from 6.37 Å to 6.43 Å (+0.94%), while the smallest diameter, identified in the rings proximate to the vacancy (highlighted in light-blue in Figure 1d), demonstrates a rise from 5.35 Å to 5.38 Å (+0.56%). A comparable trend is observed for the (6,0) SWCNT with a di-vacancy, whereby all reference rings exhibit an increase in diameter following adsorption, albeit to a lesser extent. The rings marked in red, which represent the largest diameter in this SWCNT, exhibit an increase from 5.03 Å to 5.09 Å (+1.19%), while the average diameter shows a slight rise from 4.83 Å to 4.85 Å (+0.41%); in contrast, the light-blue rings, which have the smallest diameter, demonstrate the minimal increase from 3.68 Å to 3.69 Å (+0.27%) (Figure 1b).

Table 1 presents the calculated adsorption energies and the magnetisation before and after the adsorption process for the optimised geometries, as reported for the four used functionals.

The pronounced curvature of SWCNTs markedly augments the adsorption energy, thereby facilitating more robust binding interactions between the Ni atom and the SWCNT. To illustrate, employing the identical functional, for instance, in PBE-D2 (see Table 1), the adsorption energy of Ni on pristine SWCNTs is found to be −2.16 eV for the (8,0) SWCNT and −2.63 eV for the (6,0) SWCNT. The value of the (8,0) SWCNT is in excellent agreement with the value of −2.41 and −2.4 eV obtained for the PBE-D2 functional with (5,5) and (8,0) chirality, as reported in references [27,58]. Similarly, in the case of adsorption on SWCNTs with a di-vacancy, the adsorption energy is observed to be −2.85 eV for the (8,0) SWCNT and −3.34 eV for the (6,0) SWCNT. This effect indicates that structural curvature plays a pivotal role in stabilising adsorption sites, particularly for TM atoms such as Ni.

In a previous study, Ambrusi et al. [61] investigated the deposition of Ni on multivacancy graphene using the PBE-D2 functional. The two-vacancy graphene structure was not the subject of this study; however, it was considered to be a graphene structure with four vacancies doped with a single Ni atom. This structure provides a valuable opportunity to compare the interaction of Ni with a flat carbon layer and with 2vac-(n,0) SWCNTs (n = 6,8); as in all the cases, the C atoms reconstruct, forming non-dangling bonds. Furthermore, the adsorption of Ni on vacancy-defected graphene occurs in a manner that deviates for the central location, with a C-Ni bond distance of 1.87 Å. In contrast, on the defected (n,0) SWCNT (n = 6,8), the bond distance is observed to be between 2.09 and 2.76 Å. Nevertheless, the adsorption energy was found to be −2.2 eV for the flat carbon surface (graphene), which is 0.65 eV and 1.14 eV more positive than the Ni adsorption energy on (8,0) and (6,0) SWCNTs. This phenomenon also occurs in the case of pristine graphene, where the adsorption energy of Ni is observed to be −1.75 eV. In contrast, the adsorption energy on pristine (6,0) and (8,0) SWCNT is found to be −2.63 eV and −2.16 eV, respectively.

As anticipated, the introduction of a vacancy defect results in an enhanced Ni binding affinity relative to the pristine SWCNT, when employing identical functionals and zigzag (n,0) type SWCNTs (n = 6,8). The most stable site configurations indicate that Ni binds more favourably to defective structures than to defect-free SWCNTs. To illustrate, the PBE-D2 functional (see Table 1) reveals that Ni adsorbs onto the pristine (8,0) SWCNT with an adsorption energy of −2.16 eV, while on the (8,0) SWCNT with a di-vacancy defect, the adsorption energy is −2.85 eV. Similarly, for the pristine (6,0) SWCNT, Ni exhibits an adsorption energy of −2.63 eV, while on the (6,0) SWCNT with a di-vacancy defect, this value is −3.34 eV.

In general, the assessment of dispersion energy through the utilisation of the DFT-D3 methodology has been observed to yield lower adsorption energies in comparison to the DFT-D2 approach. This reduction can be attributed to the inclusion of a repulsive term in the D3 formalism, which is absent in D2. As demonstrated in Table 1, the PBE-D3 functional yields lower adsorption energies than the PBE-D2 functional for the four systems under consideration. In addition, the binding pattern observed with the di-vacancy defect is consistently reproduced upon the inclusion of the Hubbard parameter. This trend provides further evidence that the defect has a beneficial effect on Ni adsorption, which reinforces the observed stability trends. Moreover, the majority of cases exhibit no magnetisation, with the exception of Ni adsorbed on the di-vacancy (8,0) SWCNT, as evidenced in Table 1.

The incorporation of the Hubbard parameter has been observed to enhance the adsorption energies, which can be related to the more precise treatment of localised Ni d-state. By reducing the self-interaction of d-electron, this method enhances the interaction of Ni d-states with the p-states of surface C atoms, thereby stabilising the system at lower energies. To illustrate, Table 1 presents the adsorption energy for Ni on the (8,0) SWCNT with a di-vacancy defect. Upon incorporation of the Hubbard parameter the adsorption energy for the D2 functional decreases from −2.85 eV to −3.16 eV. A similar trend is observed for the D3 functional, with a decrease from −2.71 eV to −3.02 eV; this effect is more pronounced in SWCNTs with larger diameters.

### 3.2. Electronic Structure

**Figure 2:**
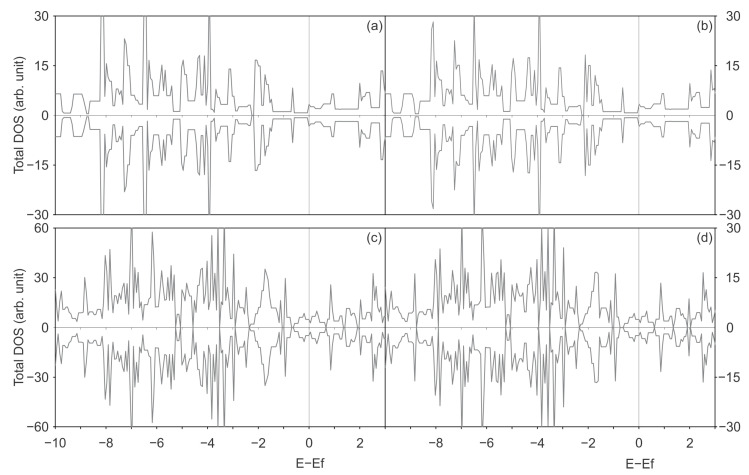
DOS of the pristine (6,0) SWCNT, calculated with (**a**) the PBE-D2 functional and (**b**) the PBE-D3 functional; and the (6,0) SWCNT with a di-vacancy defect, calculated with (**c**) the PBE-D2 functional and (**d**) the PBE-D3 functional.

**Figure 3:**
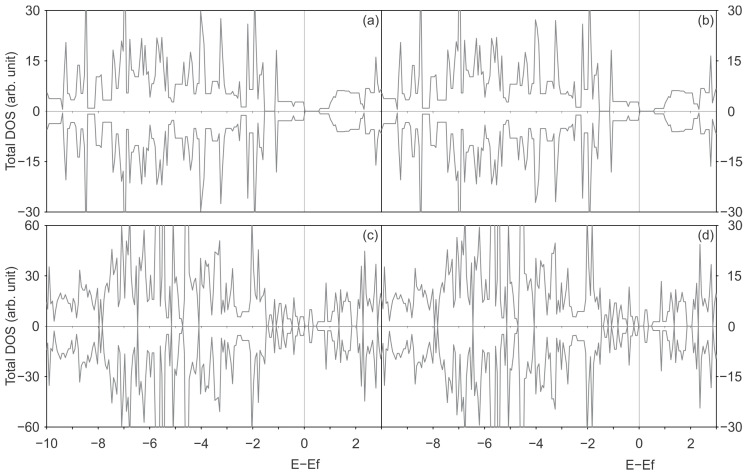
DOS of the pristine SWCNT (8,0), calculated with (**a**) the PBE-D2 functional and (**b**) the PBE-D3 functional; and the SWCNT (8,0) with a di-vacancy defect, calculated with: (**c**) the PBE-D2 functional and (**d**) the PBE-D3 functional.

A detailed examination of the electronic structure was conducted to enhance comprehension of the interaction between Ni and SWCNT and the impact of utilising a distinct functional for the dispersion and localised d-orbitals representations. Figure 2 and Figure 3 show the total DOS for pristine and defected (6,0) and (8,0) SWCNTs, respectively, with PBE-D2 and PBE-D3 functionals.

The introduction of two vacancy defects generally results in an increase in the number of states within the energy range under analysis. It is not worthy that the number of states around the Fermi level has increased. This observation may be indicative of a more reactive structure, which could prove advantageous for doping purposes. The DOS obtained with the PBE-D2 and PBE-D3 dispersion methods exhibit minimal discrepancy, which is likely attributable to the limited variation in atomic positions after relaxation with each dispersion method in the absence of Ni. 

It is noteworthy that, for the pristine (6,0) SWCNT, the number of states is not zero around the Fermi level. Matsuda et al. [62] demonstrated that the pristine (6,0) SWCNT exhibits a conductor behaviour without a band gap. The authors posit that this can be understood due to the π–π* coupling in this 3n (n = 2 … 10) zigzag SWCNT. Additionally, other research [63] suggests that for small nanotubes, the curvature is so strong that rehybridisation occurs between σ and π states, resulting in band overlap and a metallic behaviour for pristine (6,0) SWCNT.

In contrast to the pristine (6,0) SWCNT, the (8,0) SWCNT exhibits a band gap of approximately 0.53 eV for both the PBE-D2 and D3 approaches. The accuracy of this value is beyond the scope of the present work, which could prove challenging with a PBE functional. For instance, with a (9,0) chirality SWCNT, the resulting value of 0.003 eV differs from the experimental value of 0.08 eV [62]. It can be stated that, in contrast to the (6,0) chirality, the (8,0) type SWCNT presents a band gap, given that the PBE functional typically underestimates band gaps. Moreover, an increase in the number of states within the specified energy range is observed when comparing the two-vacancy (8,0) SWCNT with the pristine (8,0) SWCNT. The introduction of vacancies results in a reduction in the band gap, which is 0.13 eV for the 2vac-(8,0) SWCNT using the PBE-D2 functional. The gap remains largely unchanged when the PBE-D3 functional is employed. Additionally, the discrepancy between the DOS curves obtained with PBE-D2 and PBE-D3 remains minimal, with only slight alterations in selected peaks. Therefore, both functionals are effective in achieving the final equilibrium structure used to study the electronic structure when relaxing the SWCNT with or without defect.

**Figure 4:**
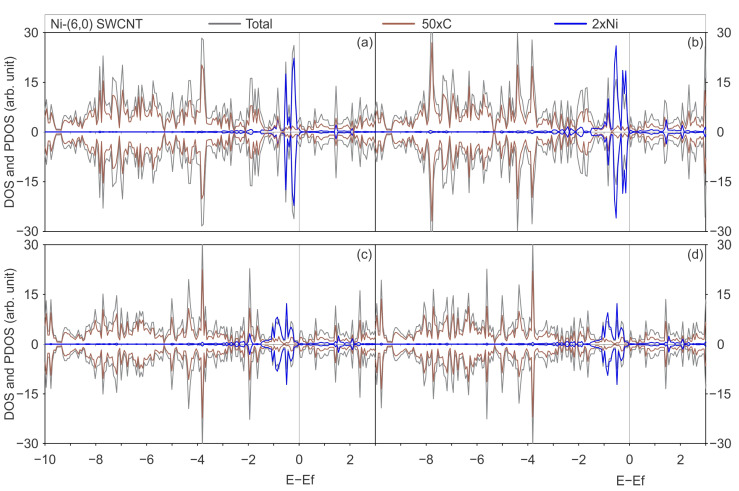
The total DOS of the most stable geometry of a Ni atom adsorbed on a pristine (6,0) SWCNT is illustrated in grey, the PDOS onto the C atoms in brown, and the PDOS onto the Ni atom in blue, calculated with (**a**) the PBE-D2 functional, (**b**) the PBE-D3 functional, (**c**) the PBE + U-D2 functional, and (**d**) the PBE + U-D3 functional.

**Figure 5:**
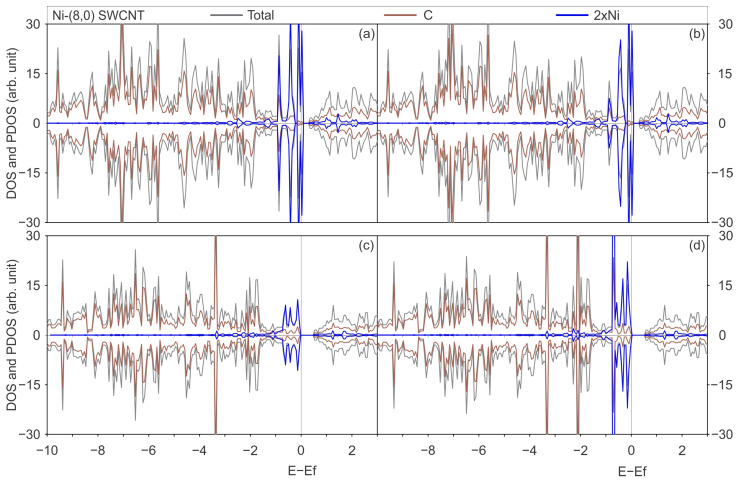
Total DOS of the most stable geometry of a Ni atom adsorbed on a pristine (8,0) SWCNT is illustrated in grey, the PDOS onto the C atoms in brown, and the PDOS onto the Ni atom in blue, calculated with (**a**) the PBE-D2 functional, (**b**) the PBE-D3 functional, (**c**) the PBE + U-D2 functional, and (**d**) the PBE + U-D3 functional.

Figure 4 and Figure 5 illustrate the total DOS and PDOS of Ni adsorbed on pristine and defective SWCNTs of (6,0) and (8,0) type, with PBE-D2 and PBE-D3 functionals with and without the U_eff_ parameter.

The DOS curves displayed on Figure 4 and Figure 5 illustrate an increase in the number of states around the Fermi level within the valence band. The observed effect can be attributed to the hybridisation between Ni and C orbitals, in the energy range between −2 and 0 eV, with a negligible contribution at energies lower than −2 eV. This hybridisation around the Fermi level was observed for Ni and other TMs interacting with other chiralities of SWCNTs because the C p and TM d states were previously reported in the literature [7,25,32]. Zhuang et al. conducted a study examining the interactions between TMs, including Ni, and defective CNTs. The same conclusion regarding hybridisation is reached by these authors through an analysis of the band structures of metal-adsorbed on pristine and defective (5,5) CNT [7]. Seenithurai et al. reached the same conclusion in the case of H_2_ adsorption on Ni and passivated Ni doped (5,0) SWCNT [25], while Mashapa and Ray analysed the case of low-concentration substitutional doping of TM on armchair (5,5) SWCNT [32].

**Figure 6:**
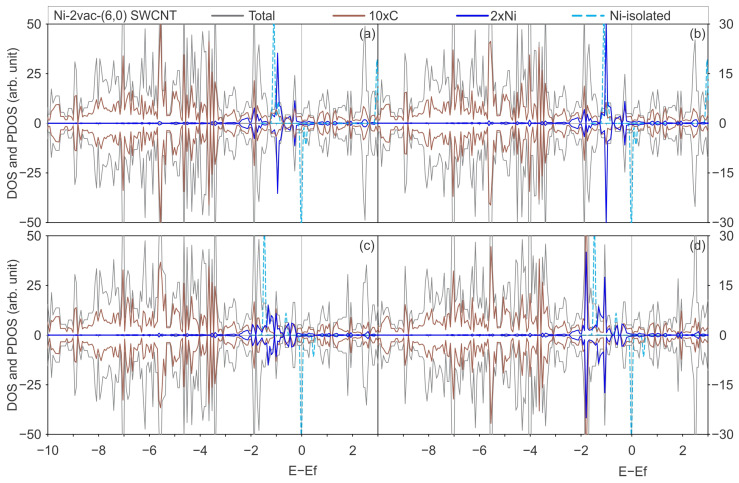
The total DOS of a Ni atom adsorbed on a (6,0) SWCNT with a di-vacancy is illustrated in grey, the PDOS onto the C atoms in brown, the PDOS onto the Ni atom in blue, and the DOS of the isolated Ni atom in light-blue dashed lines, calculated with (**a**) the PBE-D2 functional, (**b**) the PBE-D3 functional, (**c**) the PBE + U-D2 functional, and (**d**) the PBE + U-D3 functional.

**Figure 7:**
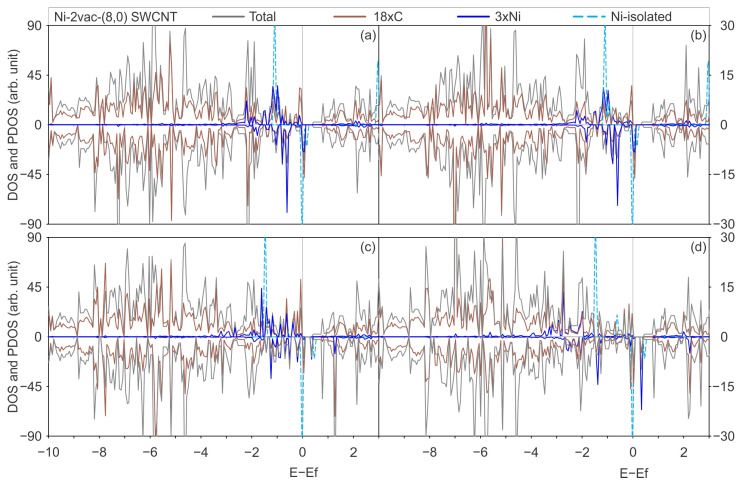
Total DOS of a Ni atom adsorbed on an (8,0) SWCNT with a di-vacancy is illustrated in grey, the PDOS onto the C atoms in brown, the PDOS onto the Ni atom in blue, and the DOS of the isolated Ni atom in light-blue dashed lines, calculated with (**a**) the PBE-D2 functional, (**b**) the PBE-D3 functional, (**c**) the PBE + U-D2 functional, and (**d**) the PBE + U-D3 functional.

Figure 6 and Figure 7 illustrate the total DOS and PDOS of Ni adsorbed on defected SWCNTs of (6,0) and (8,0) types, employing the PBE-D2 and PBE-D3 functionals with and without the U_eff_ parameter.

In general, when a Ni atom is adsorbed on a SWCNT surface with two vacancies, the overlapped projected states on Ni and C exhibit a greater degree of energy dispersion than those observed in the Ni adsorbed on pristine SWCNTs. This can be corroborated by a comparison of the PDOS on Ni and C of Ni-2vac-(6,0) SWCNT (Figure 6), which demonstrates a pronounced hybridisation between Ni and C states that are not only predominately observed in the −2 to 0 eV range as seen in the case of Ni adsorbed on pristine (6,0) SWCNT (Figure 4) but also at lower energies. Figure 6 also illustrates the presence of overlapped states between −3 and −2 eV for the Ni-2vac-(6,0) SWCNT. Moreover, the electronic states of Ni are shifted to lower energies in comparison with the states of isolated Ni, indicating enhanced stability of Ni after adsorption. Furthermore, the Ni atom electronic states are shifted to lower energies, indicating a stabilisation after adsorption. Additionally, the isolated states of the Ni atom are located at specific energy values, in contrast to adsorbed Ni, concluding that the interaction of Ni with the SWCNT surface is considerable. It is observed that the notable peak at the Fermi level of isolated Ni effectively vanishes and that numerous peaks at lower energies are superimposed with the C states ones. In addition, a comparison of the DOS and PDOS for the Ni-(6,0) SWCNT (Figure 4) and the Ni-2vac-(6,0) SWCNT (Figure 6) reveals a greater number of peaks for the latter system which are indicative of stronger interactions. Nevertheless, the adsorbed Ni exhibits states that are symmetric with respect to spin up and down, resulting in a lack of magnetisation in comparison with the asymmetric isolated Ni states, which have a magnetisation of 2 μ_B_ for the most stable triplet ground state.

The aforementioned overlap between Ni and C orbitals is responsible for the observed increase in states around the Fermi level of the total DOS for Ni-2vac-(6,0) SWCNT (Figure 6). Additionally, there is a hybridisation of the projected metal and carbon states in the conduction band, which is likely associated with the antibonding orbitals due to the interaction between these atoms. This further enhances the conductor behaviour that was also observed for pristine (6,0) SWCNT. Consequently, a potential application of this material is for low-resistance contact.

An important result to highlight is that regardless of the functional applied with or without U_eff_, the interaction of Ni with the (6,0) SWCNT type does not present a considerable variation on projected states of Ni that arise from the interactions with states of the C atom. In general, the total DOS and PDOS are very similar, with differences in the shapes of some peaks but not in the positions and appearance of new peaks.

Similarly to the previous behaviour observed for Ni-2vac-(6,0) SWCNT, the PDOS on Ni and C orbitals results in an increase in the total DOS for Ni-2vac-(8,0) SWCNT, which exhibits a higher number of peaks compared with Ni adatom on pristine (8,0) SWCNT (Figure 5). This can be observed by comparing it with Figure 7. In the case of Ni-(8,0) SWCNT, the Ni and C state peaks overlap for the first time in the energy region from −2 to 0 eV. This is a more localised phenomenon than that observed for Ni-2vac-(8,0) SWCNT, which may contribute to the observed weak interaction between Ni and C from the energetic perspective for the adsorption on pristine SWCNT. Also, the band gap is reduced in the (8,0) SWCNT due to this overlap around the Fermi level. The aggregation of Ni in pristine (8,0) SWCNT results in a reduction in the band gap, which nevertheless remains present with a value of 0.18, 0.19, 0.47, and 0.49 eV for PBE-D2, PBE-D3, PBE + U-D2, and PBE + U-D3, respectively (see Figure 5a, Figure 5b, Figure 5c and Figure 5d). The band gap of Ni-2vac-(8,0) SWCNT is approximately 0.38 up (0.25 down), 0.28 up (0.26 down), 0.38 up (0.18 down) eV, and 0.37 up (0.17 down) eV for PBE-D2, PBE-D3, PBE + U-D2, and PBE + U-D3, respectively (see Figure 7a, Figure 7b, Figure 7c and Figure 7d). Moreover, an examination of the PDOS curves of Ni and C surface on Ni-2vac-(6,0) SWCNT and Ni-2vac-(8,0) SWCNT reveals that the peaks of the latter are sharper and more localised. This observation supports the idea of a weaker adsorption energy of Ni-2vac-(8,0) SWCNT, as previously stated from adsorption energy analysis. 

In contrast to the Ni-2vac-(6,0) SWCNT, the inclusion of the U_eff_ results in alterations to the total DOS and PDOS for the PBE-D2 and PBE-D3. The total DOS for PBE-D2 and PBE-D3 exhibit minimal variation, beyond some changes that can be attributed to slight atomic positional shifts during the relaxation process. The inclusion of the Hubbard parameter results in a reduction in the band gap of the Ni-2vac-(8,0) SWCNT by 28% relative to the pristine (8,0) SWCNT, and it increases by 38% to the 2vac-(8,0) SWCNT. Moreover, a reduction of 0.1 eV is observed for Ni-(8,0) SWCNT, which can be related to the concurrent hybridisation of valence states and the clear overlap of metal and C orbitals in the conduction band at a lower energy than the peak of isolated Ni, which is approximately 0.5 eV. Another consequence of the interaction between the Ni states and the C states is the magnetisation observed in Ni adsorbed on defected (8,0) SWCNT with all the functionals applied. This effect is not observed in the defected (6,0) SWCNT. In light of these findings, the Ni doping of the defected (8,0) SWCNT represents a promising avenue for the development of spintronic devices, offering the potential to combine different conductivities (different band gaps) for spin up and down and magnetisation. The alteration of the band gap resulting from the incorporation of TM impurities was also observed in other materials. For instance, Soussi et al. [64] demonstrated that the band gap is reduced in SnO_2_ doped with Al.

Moreover, the incorporation of the Hubbard parameter enables the emergence of Ni states at lower energies. Peaks in the range between −3 and −2 eV occur with and without U_eff_, but their intensity is greater. This is likely due to the correction of the self-interaction energy accounted for in the Hartree potential as a result of localised unpaired electrons [65]. If the U_eff_ corrects for some of this spurious Coulomb repulsion, it leads to more stable orbitals that can interact at lower energies. This effect was not fully recognised for Ni-2vac-(6,0) SWCNT, potentially because its states are more dispersed in energy, as evidenced by broader peaks in the DOS and PDOS, particularly at the Fermi level, which corresponds to unpaired electron states.

**Figure 8:**
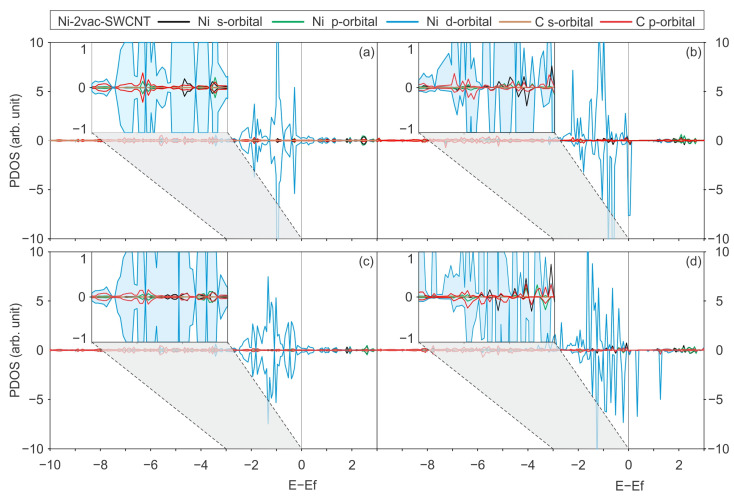
The PDOS for the orbitals of the system with a Ni atom adsorbed on a SWCNT with a di-vacancy. (6,0) SWCNT system, calculated with (**a**) the PBE-D2 functional and (**c**) the PBE + U-D2 functional. (8,0) SWCNT system, calculated with (**b**) the PBE-D2 functional and (**d**) the PBE + U-D2 functional. The colours used in the graphs represent the following orbitals: the s-orbital of Ni (black), p-orbital of Ni (green), d-orbital of Ni (blue), s-orbital of C (brown), and p-orbital of C (red). The inset at the top left of each sub-figure provides a zoomed view of the graphs for the energy region between 0 and −3 eV.

In addition to the projections on atoms, we perform the projection on orbitals of specific atoms that have been previously considered in order to establish which of these on-site contributions are the most significant. Figure 8 illustrates the results for the PBE-D2 and PBE + U-D2 functionals.

From Figure 8a,c, it can be observed that the states in the region between −3 and −2 eV for Ni-2vac-(6,0) SWCNT are predominately made up of Ni d states and C p states. A similar phenomenon is observed for Ni-2vac-(8,0) SWCNT, as shown in Figure 8b,d. However, the use of the Hubbard parameter results in an increase in these states in the region between −3 and −2 eV for Ni-2vac-(8,0) SWCNT. The participation of the d orbitals of Ni reinforces the assumption of an increase in the states in that region due to the correction of the self-interaction energy of the metal`s electrons. In the case of Ni-2vac-(6,0) SWCNT, no variation is observed for both with and without U_eff_ (see Figure 8a,c), indicating that the self-interaction is lower in this case. In both systems, close to the Fermi level and at lower energies, there is evidence of hybridisation between Ni s and localised p states with C p states. However, there is no clear hybridisation between C s states with Ni states. Compared with other contributions from p orbitals, the latter is not significant. This could be related to the fact that sp^2^ hybridisation in σ bonds is very stable.

### 3.3. Bonding Analysis

**Figure 9:**
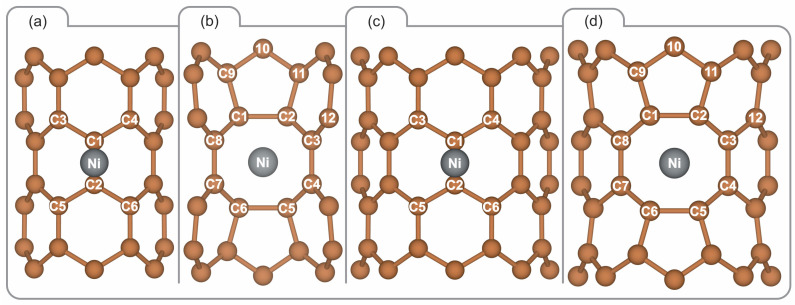
Selected atoms for bond analysis based on BO and OP values for the four systems: (**a**) Ni adsorbed on pristine (6,0) SWCNT, (**b**) Ni adsorbed on (6,0) SWCNT with a di-vacancy, (**c**) Ni adsorbed on pristine (8,0) SWCNT, and (**d**) Ni adsorbed on (8,0) SWCNT with a di-vacancy.

**Figure 10:**
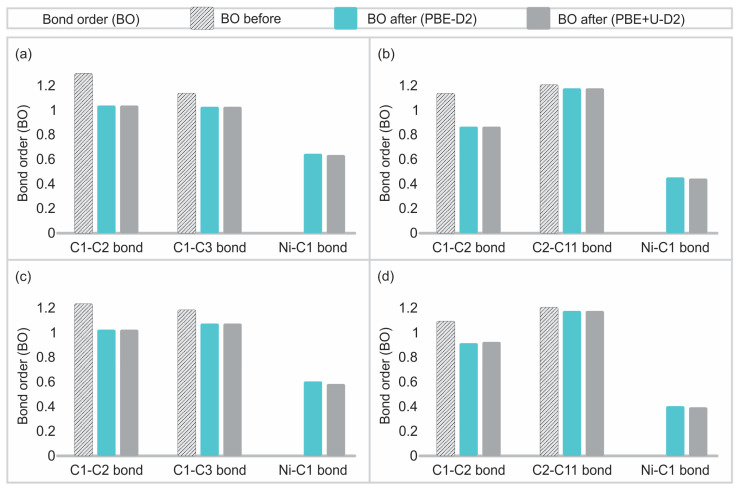
BO for selected C-C and Ni-C bonds (see Figure 9), calculated before and after Ni adsorption for the optimised systems using PBE-D2 and PBE + U-D2 functionals, for the systems: (**a**) Ni-(6,0) SWCNT, (**b**) Ni-2vac-(6,0) SWCNT, (**c**) Ni-(8,0) SWCNT, and (**d**) Ni-2vac-(8,0) SWCNT.

Table 2 presents the bond distances of the selected atoms employed for the OP and BO analysis, both before and after the Ni adsorption, with their corresponding percentage change. The OP and BO values are presented in Table S1 (Supplementary File) for both the pre- and post-adsorption states. The selected atoms for this analysis are indicated in Figure 9. Additionally, the most pertinent data from Table S1 was presented in graphical form in Figure 10, thus facilitating enhanced visualisation.

From the bond length values before and after the Ni adsorption (Table 2), it is possible to determine the variation in the bond distances for the C atoms close to the adsorption region. A significant maximum variation is detected for the SWCNT with vacancies for the (6,0) type; although, in general, they are of the same order. The observed changes in bond lengths are related to an interaction with Ni. In general, there is an increase in bond length, indicating a reduction in the strength of C-C bonds to bind with Ni. A comparison of the values the values in Table 2 with the C-C bond length far from the adsorption region (not included in the table) yields a percentage variation of 0.7%, which suggests that the effect on the structure occurs around the adsorption region.

As can be seen in Table S1 and Figure 10, the OP and BO values are not affected by the use of PBE-D2 or PBE + U-D2 functional in all the systems. This leads to the conclusion that the application of the two functionals results in a similar distribution of these chemical parameters, even in cases where the U_eff_ parameter is excluded or included. With regard to the Ni-C bond, the OP and BO are reported for only one of its nearest neighbours, given that similar values were obtained for the others. The OP and BO values for the Ni-C bond are notable, indicating the presence of a chemical bond and a higher interaction between Ni and C for the (6,0) nanotube in comparison to the (8,0) type. In contrast to the pristine SWCNTs, the Ni-C interaction is weaker for the 2vac-SWCNTs. However, the Ni atom is more stable on the SWCNT with two vacancies, and thus the coordination achieved when the Ni is adsorbed on SWCNT with two vacancies is relevant for its stability. After the adsorption of Ni, a general trend was observed whereby the closest C-C bonds to the adsorption site were found to weaken in both the pristine and defected SWCNTs. This reduction in bond strength is related to the stretching of the C-C bonds (Table 2) after adsorption. It is also noteworthy that the Ni-C bond is stronger based on these chemical parameters for the lower-diameter nanotube, which is consistent with the previous stability analysis based on the adsorption energies. For a C-C bond far from the adsorption region, the OP and BO variation is 1.2% and 1.3%, respectively. This indicates that the observed changes are due to electronic variation around the adsorption site, resulting from the interaction between Ni and the SWCNT surface.

**Figure 11:**
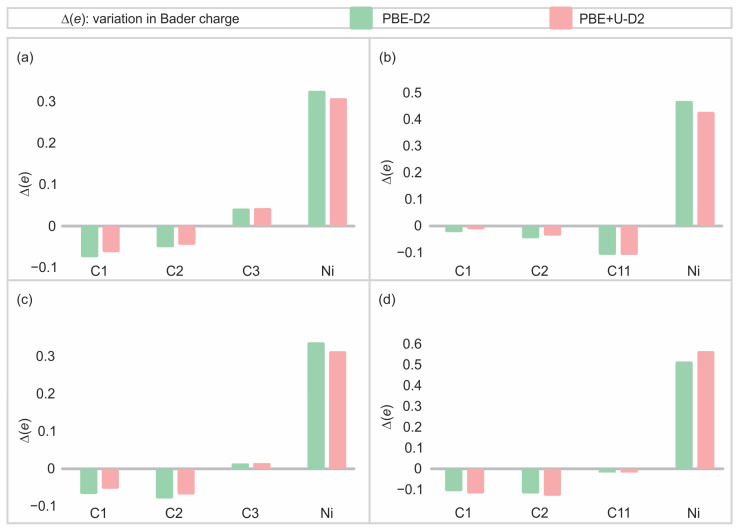
Variation in Bader charges for optimised systems on selected atoms (see Figure 9) before and after Ni adsorption using PBE-D2 and PBE + U-D2 functionals, for the systems: (**a**) Ni-(6,0) SWCNT, (**b**) Ni-2vac-(6,0) SWCNT, (**c**) Ni-(8,0) SWCNT, and (**d**) Ni-2vac-(8,0) SWCNT.

The Bader charges were obtained after Ni adsorption on pristine and defective (6,0) and (8,0) SWCNTs for the same selected atoms used for OP and BO analysis. The values are summarised in Table S2 (Supplementary File), where a positive value denotes the loss of electrons, while a negative value indicates the gain of them. Additionally, the most relevant data of Table S2 were presented in graphical form in Figure 11, thus facilitating enhanced visual representation.

The Bader charges demonstrate a charge transfer from Ni to the SWCNT in all the systems, resulting in a positive Ni atom after the adsorption. This charge transfer process increases for the (8,0) nanotube in comparison with the (6,0) type, for both pristine and defective SWCNTs. Therefore, it can be concluded that the charge transfer is not the sole stabilisation mechanism, as the (6,0) SWCNT type exhibits higher Ni adsorption energies, which contradict this effect. According to this, it is possible to infer that the electronic contribution to the stability of the Ni bond with SWCNT is related not only to a charge transfer mechanism but also to the electron sharing through the overlap between orbitals, giving a more covalent character. However, the charge transfer provides a partial explanation for the enhanced stability of Ni adsorption on 2vac-SWCNT, resulting in a greater charge transfer than the adsorption on pristine SWCNT. This is likely due to the increment in the coordination for the defected SWCNT.

After the Ni adsorption, the charge on the SWCNT is redistributed, resulting in negativity for some of the C atoms close to the Ni. Despite the fact that the charge sign of carbon atoms surrounding the adsorption site follows a similar pattern, from a quantitative point of view, this pattern is not the same for (6,0) and (8,0) nanotubes. It is noteworthy that the incorporation of the U_eff_ parameter modifies the Bader charges, making a more appreciable change for the Ni atom. Hence, the correction of the Ni self-interaction spurious repulsion energy of d electrons has an influence on Bader charges, especially on Ni. In contrast, as previously stated, this effect exhibits no appreciable sensitivity for OP and BO chemical parameters. Therefore, the self-energy interaction of Ni d electrons, as previously stated by the PDOS analysis, conducts to the dispersion of Ni d states at lower energies, which, from a stability standpoint, favourably alters the charge transfer. This effect is more pronounced for (8,0) SWCNT, because it is the most affected for the localised Ni d bands, as was also previously discussed in the PDOS analysis. It is pertinent to make a comment regarding the bonding character and Bader charge analysis in a related system. In the case of Rh adatom stability on graphene, with and without defects, Ambrusi et al. [66] evaluated the feasibility of achieving a uniform dispersion of the metallic atom. The authors found that when H_2_ molecules bind to Rh adatoms, an electrostatic interaction occurs due to a charge transfer from the metal to the graphene surfaces after adsorption. Bonding and Bader charge analysis are analogous to the CNTs in the present study.

**Figure 12:**
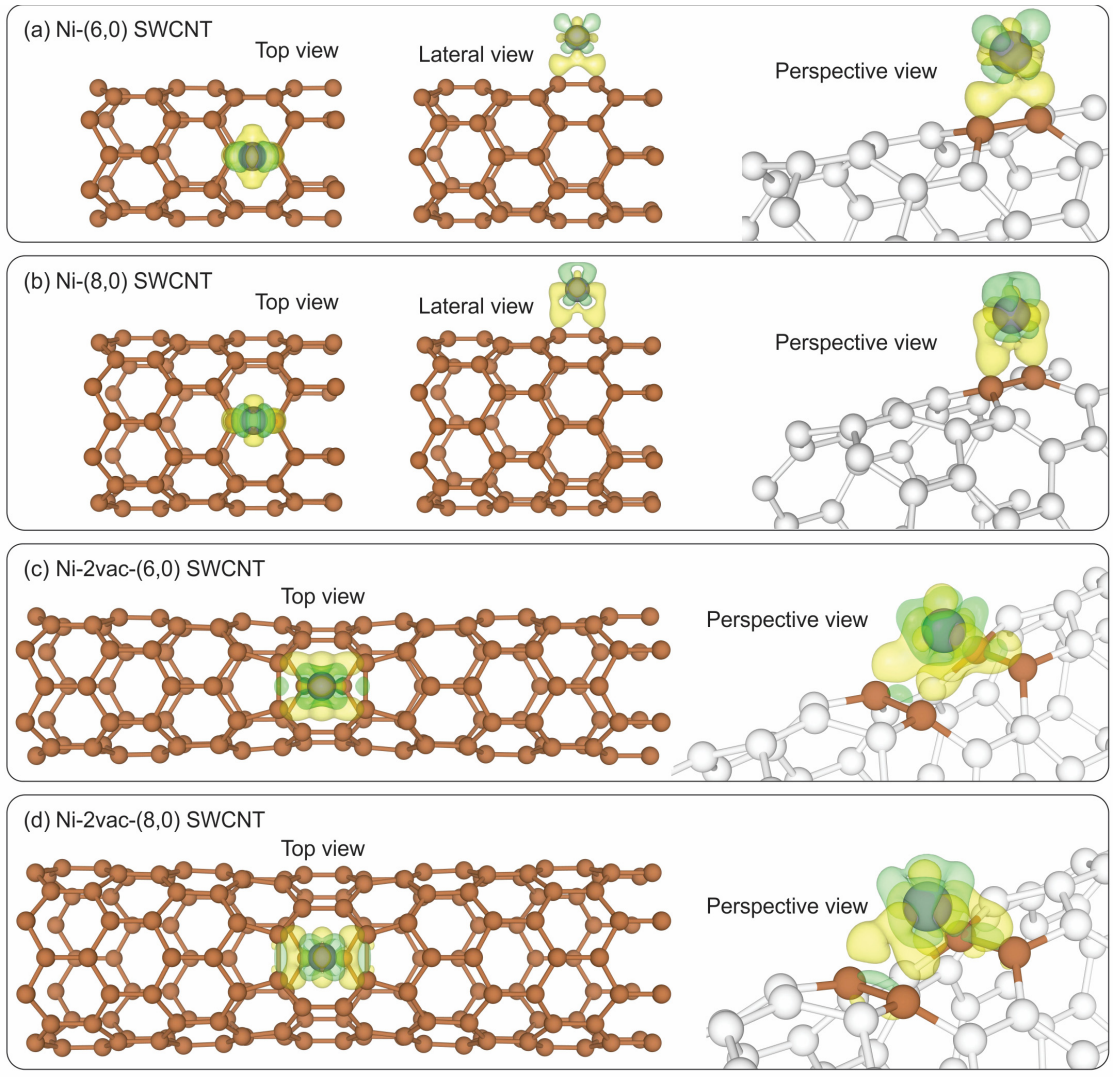
Isosurfaces of the charge density differences. Isosurface value = 0.05 e/Å^3^ (**a**,**b**) and 0.08 e/Å^3^ (**c**,**d**). Electron accumulation (positive) and depletion (negative) regions are indicated in yellow and green, respectively.

Figure 12 shows the isosurfaces of the charge density differences which complement the charge distribution analysis.

Figure 12a,b show the accumulation of electrons between Ni and C atoms of the bridge site on the (6,0) SWCNT and (8,0) SWCNT, respectively. This indicates the formation of a bond between Ni and C at this adsorption site. Similarly, in Figure 12c,d, it is possible to appreciate a symmetric distribution of the charge for the Ni adsorbed on the two vacancy-defected SWCNT for 2vac-(6,0) SWCNT and 2vac-(8,0) SWCNT, respectively. Additionally, an accumulation of electrons is observed in the regions between Ni and C atoms, which is indicative of the formation of bonds between them. The isosurfaces surrounding the Ni atoms exhibit a depletion of electrons, indicating a positive charge, as we concluded from the Bader analysis, after the charge transfer to the nanotube surface. A comparison of the Ni-2vac-SWCNT with Ni-pristine-SWCNT enables the identification of the primary coordination in the systems with two vacancies, which are fourth-coordinated based on the accumulation of electrons between Ni and C atoms at the defect site. This fourth coordination is associated with Ni-C bonds arranged symmetrically.

## 4. Conclusions

The adsorption of the Ni atom on the di-vacancy SWCNT shows greater stability than the adsorption on pristine SWCNT. This stability increases with the tube curvature, for zigzag (n,0) nanotubes. According to the DOS, this occurs because of the presence of a larger number of states in the (6,0)-type nanotube compared to (8,0), which are additionally more overlapped, forming broader bands. This allows for more dispersed energy hybridisation between PDOS on Ni and C, forming not only an overlap of them at lower energies but also bands distributed across a wider energy range, instead of being localised at specific energy values. This mechanism allows for stronger interaction and higher stability for Ni adsorption. Although the overlap between C orbitals of defected SWCNT and the Ni orbitals occurs almost in the same energy range comparing the 2vac-(6,0) SWCNT and 2vac-(8,0) SWCNT structures, the wider overlapped peaks in the former introduce additional stability. In pristine SWCNTs, the number of states is lower, and the overlap between Ni and C states is concentrated in the −2 and 0 eV range. In vacancy-defected SWCNTs, an additional significant hybridisation occurs at lower energies between −3 and −2 eV. This overlap is mainly between Ni d states and C p states. A comparison of the different functionals reveals that the electronic structure is not affected by the use of PBE-D2 and PBE-D3. The introduction of the U_eff_ parameter results in more stable adsorption energies, making this effect more pronounced for the (8,0) SWCNT. There is a charge transfer from Ni to the substrate that contributes more considerably when vacancy defects are included, having determinant participation in the stability. However, for the defected SWCNT, the (8,0)-type nanotube presents a larger charge transfer than the (6,0) configuration, thereby forming a more ionic bond. In the case of the (6,0) nanotube, the Ni strongly interacts with C through the overlap of the states that share electrons. The bonding analysis between Ni and C on SWCNT reveals that the OP and BO are not higher for the defect. Consequently, the stability and the number of states interacting correspond to a more coordinated bond, which is verified through OP, BO, and the geometry of the adsorption site.

## Figures and Tables

**Table 1:** The adsorption energy (*E_ads_*/eV) and magnetisation (μ_B_) values for the four Ni-SWCNT systems studied before and after the adsorption process, with all used functionals. The symbol ‘U’ denotes the Hubbard parameter.

System	Functional	*E_ads_*/eV	μ_B_ (After) †
Ni-(6,0) SWCNT: Adsorbed Ni atom on the pristine (6,0) SWCNT	PBE-D2	−2.63	0.0
	PBE-D3	−2.54	0.0
	PBE + U-D2	−2.79	0.0
	PBE + U-D3	−2.70	0.0
Ni-(8,0) SWCNT: Adsorbed Ni atom on the pristine (8,0) SWCNT	PBE-D2	−2.16	0.0
	PBE-D3	−2.01	0.3
	PBE + U-D2	−2.34	0.0
	PBE + U-D3	−2.25	0.0
Ni-2vac-(6,0) SWCNT: Adsorbed Ni atom on the (6,0) SWCNT with a di-vacancy	PBE-D2	−3.34	0.0
	PBE-D3	−3.20	0.0
	PBE + U-D2	−3.41	0.0
	PBE + U-D3	−3.27	0.0
Ni-2vac-(8,0) SWCNT: Adsorbed Ni atom on the (8,0) SWCNT with a di-vacancy	PBE-D2	−2.85	1.4
	PBE-D3	−2.71	1.3
	PBE + U-D2	−3.16	1.5
	PBE + U-D3	−3.02	1.3

† μ_B_ (before adsorption) = 0.

**Table 2:** Bond distance values for selected C-C and Ni-C bonds (see Figure 9) before and after Ni adsorption for the four systems.

System	Bond	Distance ^1^ [Å]	Change ^2^
Ni-(6,0) SWCNT: Adsorbed Ni atom on the pristine (6,0) SWCNT	C1-C2	1.45 (1.40)	+3.5%
	C1-C3	1.44 (1.48)	+2.7%
	Ni-C1	1.89	-
	C1-C2	1.45 (1.41)	+2.8%
Ni-(8,0) SWCNT: Adsorbed Ni atom on the pristine (8,0) SWCNT	C1-C3	1.46 (1.43)	+2.0%
	Ni-C1	1.90	-
	C1-C2	1.54 (1.45)	+6.2%
	C2-C11	1.44 (1.42)	+1.4%
Ni-2vac-(6,0) SWCNT: Adsorbed Ni atom on the (6,0) SWCNT with a di-vacancy	C2-C3	1.45 (1.44)	+0.7%
	C3-C4	1.39 (1.40)	−0.7%
	C3-C12	1.42 (1.44)	−1.4%
	Ni-C1	2.04	-
Ni-2vac-(8,0) SWCNT: Adsorbed Ni atom on the (8,0) SWCNT with a di-vacancy	C1-C2	1.52 (1.47)	+3.4%
	C2-C11	1.44 (1.42)	+1.4%
	C2-C3	1.45 (1.42)	+2.1%
	C3-C4	1.41 (1.43)	−1.4%

^1^ In brackets are the distances before the adsorption process. ^2^ (+) elongation, (−) contraction.

## Data Availability

The raw data supporting the conclusions of this article will be made available by the authors on request due to the size of the electron files.

## Supplementary Materials

The following supporting information can be downloaded at https://www.mdpi.com/article/10.3390/ma17246236/s1, Table S1: Bond order (BO) and overlap population (OP) values for selected C-C and Ni-C bonds (see Figure 9), measured before and after Ni adsorption for the four systems; Table S2: Bader charges for the optimised systems before and after the Ni atom adsorption using PBE-D2 and PBE+U-D2 functionals. Where ∆ indicates the variation in Bader charge following the adsorption process.

## References

[1] Ma P.C., Siddiqui N.A., Marom G., Kim J.K. (2010). Dispersion and Functionalization of Carbon Nanotubes for Polymer-Based Nanocomposites: A Review. Compos. Part A Appl. Sci. Manuf..

[2] Soni S.K., Thomas B., Kar V.R. (2020). A Comprehensive Review on CNTs and CNT-Reinforced Composites: Syntheses, Characteristics and Applications. Mater. Today Commun..

[3] Liu X.M., Huang Z.D., Oh S.W., Zhang B., Ma P.C., Yuen M.M.F., Kim J.K. (2012). Carbon Nanotube (CNT)-Based Composites as Electrode Material for Rechargeable Li-Ion Batteries: A Review. Compos. Sci. Technol..

[4] Yusfi M., Jonuarti R., Wungu T.D.K., Suprijadi (2021). Density Functional Theory of Ni-Doped (10, 0) Single-Walled Carbon Nanotubes for C_2_H_2_ and C_2_H_4_ Sensing. J. Phys. Conf. Ser..

[5] Lilloja J., Kibena-Põldsepp E., Sarapuu A., Douglin J.C., Käärik M., Kozlova J., Paiste P., Kikas A., Aruväli J., Leis J. (2021). Transition-Metal- And Nitrogen-Doped Carbide-Derived Carbon/Carbon Nanotube Composites as Cathode Catalysts for Anion-Exchange Membrane Fuel Cells. ACS Catal..

[6] Ramirez-De-arellano J.M., Canales M., Magaña L.F. (2021). Carbon Nanostructures Doped with Transition Metals for Pollutant Gas Adsorption Systems. Molecules.

[7] Zhuang H.L., Zheng G.P., Soh A.K. (2008). Interactions between Transition Metals and Defective Carbon Nanotubes. Comput. Mater. Sci..

[8] Andriotis A.N., Menon M., Froudakis G.E. (2000). Various Bonding Configurations of Transition-Metal Atoms on Carbon Nanotubes: Their Effect on Contact Resistance. Appl. Phys. Lett..

[9] Abbasi M., Nemati-Kande E. (2021). Enhancing the Reactivity of Carbon-Nanotube for Carbon Monoxide Detection by Mono- and Co-Doping of Boron and Nitrogen Heteroatoms: A DFT and TD-DFT Study. J. Phys. Chem. Solids.

[10] Bazmi M., Askari S., Ghasemy E., Rashidi A., Ettefaghi E. (2019). Nitrogen-Doped Carbon Nanotubes for Heat Transfer Applications: Enhancement of Conduction and Convection Properties of Water/N-CNT Nanofluid. J. Therm. Anal. Calorim..

[11] Mousavi-Khoshdel S.M., Jahanbakhsh-bonab P., Targholi E. (2016). Structural, Electronic Properties, and Quantum Capacitance of B, N and P-Doped Armchair Carbon Nanotubes. Phys. Lett. Sect. A Gen. At. Solid State Phys..

[12] Zhang S., Nguyen N., Leonhardt B., Jolowsky C., Hao A., Park J.G., Liang R. (2019). Carbon-Nanotube-Based Electrical Conductors: Fabrication, Optimization, and Applications. Adv. Electron. Mater..

[13] An W., Turner C.H. (2009). Chemisorption of Transition-Metal Atoms on Boron- And Nitrogen-Doped Carbon Nanotubes: Energetics and Geometric and Electronic Structures. J. Phys. Chem. C.

[14] Ao C., Zhao W., Ruan S., Qian S., Liu Y., Wang L., Zhang L. (2020). Theoretical Investigations of Electrochemical CO_2_ Reduction by Transition Metals Anchored on CNTs. Sustain. Energy Fuels.

[15] Liu Y., Jiang H., Zhu Y., Yang X., Li C. (2016). Transition Metals (Fe, Co, and Ni) Encapsulated in Nitrogen-Doped Carbon Nanotubes as Bi-Functional Catalysts for Oxygen Electrode Reactions. J. Mater. Chem. A.

[16] Zhang X., Cui H., Chen D., Dong X., Tang J. (2018). Electronic Structure and H_2_S Adsorption Property of Pt_3_ Cluster Decorated (8, 0) SWCNT. Appl. Surf. Sci..

[17] Zuo T., Li J., Gao Z., Wu Y., Zhang L., Da B., Zhao X., Xiao L. (2020). Simultaneous Improvement of Electrical Conductivity and Mechanical Property of Cr Doped Cu/CNTs Composites. Mater. Today Commun..

[18] González Fá A.J., Orazi V., González E.A., Juan A., López-Corral I. (2017). DFT Study of β-D-Glucose Adsorption on Single-Walled Carbon Nanotubes Decorated with Platinum. A Bonding Analysis. Appl. Surf. Sci..

[19] González Fá A.J., Orazi V., Jasen P., Marchetti J.M., López-Corral I. (2020). Adsorption of Carbonyl Sulfide on Pt-Doped Vacancy-Defected SWCNT: A DFT Study. Appl. Surf. Sci..

[20] Gui Y., Zhang X., Lv P., Wang S., Tang C., Zhou Q. (2018). Ni-CNT Chemical Sensor for SF_6_ Decomposition Components Detection: A Combined Experimental and Theoretical Study. Sensors.

[21] Zhang X., Gong X. (2015). DFT, QTAIM, and NBO Investigations of the Ability of the Fe or Ni Doped CNT to Absorb and Sense CO and NO. J. Mol. Model..

[22] Lu J., Zhang X., Wu X., Dai Z., Zhang J. (2015). A Ni-Doped Carbon Nanotube Sensor for Detecting Oil-Dissolved Gases in Transformers. Sensors.

[23] Li X., Liu L., Wang M., Wang Z. (2016). Adsorption and Dissociation of O_2_ on Ni-Doped (5, 5) SWCNT: A DFT Study. Appl. Surf. Sci..

[24] Nguyen T.T.H., Le V.K., Le Minh C., Nguyen N.H. (2017). A Theoretical Study of Carbon Dioxide Adsorption and Activation on Metal-Doped (Fe, Co, Ni) Carbon Nanotube. Comput. Theor. Chem..

[25] Seenithurai S., Kodi Pandyan R., Vinodh Kumar S., Mahendran M. (2013). H_2_ Adsorption in Ni and Passivated Ni Doped 4 Å Single Walled Carbon Nanotube. Int. J. Hydrogen Energy.

[26] Zhao S., Wang T., Zhou G., Zhang L., Lin C., Veder J.P., Johannessen B., Saunders M., Yin L., Liu C. (2020). Controlled One-Pot Synthesis of Nickel Single Atoms Embedded in Carbon Nanotube and Graphene Supports with High Loading. ChemNanoMat.

[27] Xiao L., Chu W., Sun W., Xue Y., Jiang C. (2017). Enhancement of Hydrogen Sorption on Metal(Ni, Rh, Pd) Functionalized Carbon Nanotubes: A DFT Study. Chem. Res. Chin. Univ..

[28] Li W., Lu X.M., Li G.Q., Ma J.J., Zeng P.Y., Chen J.F., Pan Z.L., He Q.Y. (2016). First-Principle Study of SO_2_ Molecule Adsorption on Ni-Doped Vacancy-Defected Single-Walled (8,0) Carbon Nanotubes. Appl. Surf. Sci..

[29] Li W., Ma J.J., Liu P., Pan Z.L., He Q.Y. (2015). First-Principles Study of the Adsorption Sensitivity of Ni-Doped Single-Walled Zigzag (n,0)CNTs (n = 4,5,6) toward SO_2_ Molecules. Appl. Surf. Sci..

[30] Demir S., Fellah M.F. (2020). Carbon Nanotubes Doped with Ni, Pd and Pt: A Density Functional Theory Study of Adsorption and Sensing NO. Surf. Sci..

[31] Zhang X., Gui Y., Xiao H., Zhang Y. (2016). Analysis of Adsorption Properties of Typical Partial Discharge Gases on Ni-SWCNTs Using Density Functional Theory. Appl. Surf. Sci..

[32] Mashapa M.G., Ray S.S. (2010). DFT Studies of Low Concentration Substitutional Doping of Transition-Metals on Single-Walled Carbon Nanotube Surface. J. Nanosci. Nanotechnol..

[33] Chen Y.K., Liu L.V., Tian W.Q., Wang Y.A. (2011). Theoretical Studies of Transition-Metal-Doped Single-Walled Carbon Nanotubes. J. Phys. Chem. C.

[34] Yagi Y., Briere T.M., Sluiter M.H.F., Kumar V., Farajian A.A., Kawazoe Y. (2004). Stable Geometries and Magnetic Properties of Single-Walled Carbon Nanotubes Doped with 3d Transition Metals: A First-Principles Study. Phys. Rev. B Condens. Matter Mater. Phys..

[35] Aghashiri A., Fotooh F.K., Hashemian S. (2019). Density Functional Calculations of Nickel, Palladium and Cadmium Adsorption onto (10,0) Single-Walled Carbon Nanotube. J. Mol. Model..

[36] Dasilva S., López-Planes R. (2017). Electronic Study of Carbon Nanotube (6,0) Doped with Transition Metals: Copper, Silver and Gold. J. Comput. Methods Sci. Eng..

[37] Luna C.R., Bechthold P., Brizuela G., Juan A., Pistonesi C. (2018). The Adsorption of CO, O_2_ and H_2_ on Li–Doped Defective (8,0) SWCNT: A DFT Study. Appl. Surf. Sci..

[38] Patrignani M., Juan J., Nagel O., Reimers W., Luna R., Jasen P. (2024). V The Adsorption of CO and NO on (8,0) SWCNT Decorated with Transition Metals: A DFT Study as a Possible Gas Sensor. Powder Technol..

[39] Cui H., Zhang X., Chen D., Tang J. (2019). Pt & Pd Decorated CNT as a Workable Media for SOF_2_ Sensing: A DFT Study. Appl. Surf. Sci..

[40] Chiral Data up to (40,40). https://www.photon.t.u-tokyo.ac.jp/~maruyama/kataura/chiraldata.html.

[41] Jia G., Li L., Wu T., Wang X., An S. (2016). Curvature, Vacancy Size and Chirality Effects of Mono- to Octa-Vacancies in Zigzag Single-Walled Carbon Nanotubes. New J. Chem..

[42] Kresse G., Furthmüller J. (1996). Efficient Iterative Schemes for Ab Initio Total-Energy Calculations Using a Plane-Wave Basis Set. Phys. Rev. B.

[43] Kresse G., Furthmüller J. (1996). Efficiency of Ab-Initio Total Energy Calculations for Metals and Semiconductors Using a Plane-Wave Basis Set. Comput. Mater. Sci..

[44] Perdew J.P., Burke K., Ernzerhof M. (1996). Generalized Gradient Approximation Made Simple. Phys. Rev. Lett..

[45] Blöchl P.E. (1994). Projector Augmented-Wave Method. Phys. Rev. B.

[46] Kresse G., Joubert D. (1999). From Ultrasoft Pseudopotentials to the Projector Augmented-Wave Method. Phys. Rev. B.

[47] Monkhorst H.J., Pack J.D. (1976). Special Points for Brillouin-Zone Integrations. Phys. Rev. B.

[48] Press W.H. (1989). Numerical Recipes in Pascal: The Art of Scientific Computing.

[49] Grimme S. (2006). Semiempirical GGA-Type Density Functional Constructed with a Long-Range Dispersion Correction. J. Comput. Chem..

[50] Grimme S., Antony J., Ehrlich S., Krieg H. (2010). A Consistent and Accurate Ab Initio Parametrization of Density Functional Dispersion Correction (DFT-D) for the 94 Elements H-Pu. J. Chem. Phys..

[51] Dudarev S.L., Botton G.A., Savrasov S.Y., Humphreys C.J., Sutton A.P. (1998). Electron-Energy-Loss Spectra and the Structural Stability of Nickel Oxide: An LSDA+U Study. Phys. Rev. B Condens. Matter Mater. Phys..

[52] Ao B. (2019). Atom-Resolved Chemical States in the Multivalent U-TM-O (TM: Ti, V, Cr, Mn, Fe, Ni, Nb, Mo, W) Ternary Oxides from First-Principles. J. Phys. Chem. C.

[53] Piotrowski M.J., Ungureanu C.G., Tereshchuk P., Batista K.E.A., Chaves A.S., Guedes-Sobrinho D., Da Silva J.L.F. (2016). Theoretical Study of the Structural, Energetic, and Electronic Properties of 55-Atom Metal Nanoclusters: A DFT Investigation within van Der Waals Corrections, Spin−Orbit Coupling, and PBE+U of 42 Metal Systems. J. Phys. Chem. C.

[54] Bader R.F.W. (1990). Atoms in Molecules. A Quantum Theory.

[55] Manz T.A., Limas N.G. Chargemol Program for Performing DDEC Analysis 2016. https://sourceforge.net/projects/ddec/files/.

[56] Manz T.A., Limas N.G. (2016). Introducing DDEC6 Atomic Population Analysis: Part 1. Charge Partitioning Theory and Methodology. RSC Adv..

[57] Limas N.G., Manz T.A. (2016). Introducing DDEC6 Atomic Population Analysis: Part 2. Computed Results for a Wide Range of Periodic and Nonperiodic Materials. RSC Adv..

[58] Durgun E., Dag S., Bagci V.M.K., Gülseren O., Yildirim T., Ciraci S. (2003). Systematic Study of Adsorption of Single Atoms on a Carbon Nanotube. Phys. Rev. B Condens. Matter Mater. Phys..

[59] Gaztañaga F., Sandoval M.G., Luna C.R., Jasen P.V. (2020). Theoretical Study about Alkali Metal Adsorption on Pristine and Defective (8,0) SWCNT: Geometrical, Magnetic and Electronic Changes. Appl. Surf. Sci..

[60] Luna C.R., Verdinelli V., Germán E., Seitz H., Volpe M.A., Pistonesi C., Jasen P.V. (2015). Hydrogen Adsorption and Associated Electronic and Magnetic Properties of Rh-Decorated (8,0) Carbon Nanotubes Using Density Functional Theory. J. Phys. Chem. C.

[61] Ambrusi R.E., Orazi V., Marchetti J.M., Pronsato M.E. (2020). Ni Clusters Embedded in Multivacancy Graphene Substrates. J. Phys. Chem. Solids.

[62] Matsuda Y., Tahir-Kheli J., Goddard W.A. (2010). Definitive Band Gaps for Single-Wall Carbon Nanotubes. J. Phys. Chem. Lett..

[63] Charlier J.C., Blase X., Roche S. (2007). Electronic and Transport Properties of Nanotubes. Rev. Mod. Phys..

[64] Soussi A., Haounati R., Ait hssi A., Taoufiq M., Baoubih S., Jellil Z., El Hankari S., Elfanaoui A., Markazi R., Ihlal A. (2024). Investigating Structural, Morphological, Electronic, and Optical Properties of SnO_2_ and Al-Doped SnO_2_: A Combined DFT Calculation and Experimental Study. Phys. B Condens. Matter.

[65] Sholl D.S., Steckel J.A. (2009). Density Functional Theory. A Practical Introduction.

[66] Ambrusi R.E., Luna C.R., Juan A., Pronsato M.E. (2016). DFT Study of Rh-Decorated Pristine, B-Doped and Vacancy Defected Graphene for Hydrogen Adsorption. RSC Adv..

