# Supplementary material for: DFT Study of the Stability and Electronic Properties of Ni-Doped Defected (6,0) and (8,0) Single-Walled Carbon Nanotubes
Source: Materials (Basel). 2024 Dec 20;17(24):6236. doi: 10.3390/ma17246236 (PMC11677741; doi:10.3390/ma17246236)
Supplement: Supplementary file 1 [file materials-17-06236-s001.zip › materials-3353062-supplementary.pdf]

materials

MDPI

Supplementary Material

Table S1. Bond order (BO) and overlap population (OP) values for selected C-C and Ni-C bonds (see Figure 9), measured before and after Ni adsorption for the four systems.

System	Bond	BO (PBE-D2)		BO (PBE+U-D2)		OP (PBE-D2)		OP (PBE+U-D2)	
		After (before)	%Δ	After (before)	%Δ	After (before)	%Δ	After (before)	%Δ
**Ni-(6,0) SWCNT**	C1-C2	1.03 (1.29)	-20.3	1.03 (1.29)	-20.1	0.71 (0.83)	-14.6	0.71 (0.83)	-14.6
	C1-C3	1.02 (1.13)	-9.6	1.02 (1.13)	-9.5	0.69 (0.74)	-6.8	0.69 (0.74)	-6.8
	Ni-C1	0.64	-	0.63	-	0.48	-	0.47	-
**Ni-(8,0) SWCNT**	C1-C2	1.02 (1.23)	-17.0	1.02 (1.23)	-16.9	0.70 (0.80)	-11.7	0.70 (0.80)	-11.8
	C1-C3	1.07 (1.18)	-9.1	1.07 (1.18)	-9.0	0.72 (0.76)	-6.3	0.72 (0.76)	-6.3
	Ni-C1	0.60	-	0.58	-	0.45	-	0.44	-
**Ni-2vac-(6,0) SWCNT**	C1-C2	0.86 (1.13)	-24.2	0.86 (1.13)	-24.3	0.60 (0.74)	-19.0	0.60 (0.74)	-19.3
	C2-C11	1.17 (1.20)	-2.4	1.17 (1.20)	-2.5	0.77 (0.78)	-1.3	0.77 (0.78)	-1.4
	C2-C3	1.11 (1.16)	-4.4	1.11 (1.16)	-4.4	0.74 (0.76)	-2.5	0.74 (0.76)	-2.5
	C3-C4	1.27 (1.29)	-1.6	1.27 (1.29)	-1.5	0.82 (0.83)	-0.8	0.82 (0.83)	-0.7
	C3-C12	1.16 (1.13)	+3.0	1.16 (1.13)	+3.1	0.76 (0.74)	+2.9	0.76 (0.74)	+2.9
	Ni-C1	0.45	-	0.44	-	0.34	-	0.34	-
**Ni-2vac-(8,0) SWCNT**	C1-C2	0.91 (1.09)	-16.0	0.92 (1.09)	-15.6	0.63 (0.71)	-11.6	0.63 (0.71)	-11.3
	C2-C11	1.17 (1.20)	-3.0	1.17 (1.20)	-2.6	0.77 (0.78)	-1.8	0.77 (0.78)	-1.5
	C2-C3	1.15 (1.24)	-7.7	1.15 (1.24)	-7.2	0.76 (0.80)	-5.4	0.76 (0.80)	-5.1
	C3-C4	1.24 (1.19)	+4.5	1.24 (1.19)	+4.8	0.80 (0.77)	+4.6	0.81 (0.77)	+4.9
	C3-C12	1.19 (1.16)	+2.4	1.19 (1.16)	+2.5	0.77 (0.76)	+2.4	0.77 (0.76)	+2.4
	Ni-C1	0.40	-	0.39	-	0.31	-	0.30	-

Table S2. Bader charges for the optimised systems before and after the Ni atom adsorption using PBE-D2 and PBE+U-D2 functionals. Where Δ indicates the variation in Bader charge following the adsorption process.

System	Atom		(PBE-D2)		(PBE+U-D2)	
		**Before (*e*)**	**After (*e*)**	**Δ(*e*)**	**After (*e*)**	**Δ(*e*)**
Ni-(6,0) SWCNT	C1	-0.013	-0.088	-0.075	-0.077	-0.063
	C2	0.002	-0.049	-0.051	-0.043	-0.045
	C3	0.013	0.055	0.042	0.056	0.043
	Ni		0.326	0.326	0.308	0.308
Ni-(8,0) SWCNT	C1	0.004	-0.064	-0.067	-0.050	-0.053
	C2	0.011	-0.067	-0.079	-0.057	-0.068
	C3	0.004	0.019	0.014	0.019	0.015
	Ni		0.338	0.338	0.314	0.314
Ni-2vac-(6,0) SWCNT	C1	-0.146	-0.169	-0.023	-0.158	-0.012
	C2	0.019	-0.027	-0.046	-0.017	-0.036
	C3	-0.001	0.008	0.009	0.008	0.009
	C11	0.074	-0.033	-0.108	-0.034	-0.108
	C4	0.056	0.059	0.003	0.059	0.002
	C12	-0.010	-0.032	-0.022	-0.032	-0.022
	Ni		0.469	0.469	0.428	0.428
Ni-2vac-(8,0) SWCNT	C1	-0.095	-0.201	-0.107	-0.213	-0.118
	C2	0.060	-0.058	-0.118	-0.069	-0.129
	C3	-0.033	-0.006	0.026	-0.006	0.027
	C11	0.012	-0.005	-0.017	-0.005	-0.017
	C4	0.013	0.053	0.040	0.053	0.040
	C12	-0.019	-0.019	0.000	-0.022	-0.004
	Ni		0.516	0.516	0.566	0.566
